# Exploring Attentional Mechanisms of Strategic Self-Talk Through Heart-Rate Variability in a Golf-Putting Task Among Novices

**DOI:** 10.3390/bs16081347

**Published:** 2026-08-05

**Authors:** Emmanouil Tzormpatzakis, Theodoros Proskinitopoulos, Orestis Panoulas, Evangelos Galanis, Evgenia Nikolakopoulou, Nikos Comoutos, Yannis Theodorakis, Antonis Hatzigeorgiadis

**Affiliations:** Department of Physical Education and Sport Science, University of Thessaly, 421 00 Trikala, Greece; etzormpatz@uth.gr (E.T.); egalanis@uth.gr (E.G.); nzourba@uth.gr (N.C.); theodorakis@uth.gr (Y.T.)

**Keywords:** attention, psychophysiology, self-regulation, sport, skill acquisition

## Abstract

The positive impact of strategic self-talk on performance in various sports tasks has been well-documented. Contemporary research has therefore started to explore the potential mechanisms through which self-talk affects performance. The primary purpose of the present study was to examine aspects of the attentional mechanisms underlying performance on a golf putting task, endorsing a psychophysiological perspective through the assessment of Heart-Rate Variability (HRV). Participants were 40 male sport science students, with no prior experience in golf putting, who were randomly assigned to control and experimental groups. The experiment was completed over four sessions, including baseline assessment, two training sessions, and final assessment. Participants of both groups followed the same training protocol, with the experimental group practicing strategic self-talk and developing personal self-talk plans for the final assessment. Performance and HRV were recorded during the baseline and final assessments. Repeated measures analysis of variance showed that the experimental group showed greater performance improvement from baseline to final assessment and superior performance compared to the control group at the final assessment. Analysis of the HRV data provided indications that the experimental group showed different patterns of Root Mean Square of Successive Differences (RMSSD) activation at the final assessment, showing a greater activation of the parasympathetic nervous system, in particular towards the latest stages of the golf-putting task. The HRV findings, which should be interpreted with caution due to the lack of multivariate significance, are in line with an attentional interpretation of self-talk effectiveness indicating a less effortful processing.

## 1. Introduction

Self-talk has been identified as a pivotal process for regulating behavior, particularly in the context of sport performance. A contemporary conceptualization (Latinjak et al., 2023) has identified two distinct self-talk entities, organic and strategic self-talk. On the one hand, organic self-talk refers to self-talk generated naturally from within the person; this can be either unintentional, less controlled, effortless, and emotionally charged self-verbalizations, which raise awareness of psychological experiences (spontaneous or System 1 processed self-talk; Latinjak et al., 2019; Van Raalte et al., 2016), or intentional, conscious, slower, and effortful, which serves to understand a situation, self-regulate, and make progress towards a goal (goal-directed or System 2 processed self-talk; Latinjak et al., 2019; Van Raalte et al., 2016). On the other hand, strategic self-talk involves the implementation of predetermined cue words or phrases conveying a message, which aim to trigger appropriate actions towards self-regulation and performance enhancement (Latinjak et al., 2019). Two major types of strategic self-talk have been identified, based on the functions they serve: instructional self-talk that aims at providing technical or tactical directions, and motivational self-talk that aims at enhancing motivation and confidence.

Strategic self-talk in sport has experienced a notable expansion, driven by its numerous practical implications, through the implementation of interventions for skill acquisition and performance-enhancement. Foundational reviews (e.g., Hardy et al., 2009; Tod et al., 2011) and a meta-analysis (Hatzigeorgiadis et al., 2011) have provided compelling evidence for the effectiveness of the strategic self-talk interventions. However, research identifying the factors governing self-talk effectiveness highlights the necessity for a comprehensive examination of the fundamental mechanisms underlying the strategic self-talk-performance relationship. Such an examination would facilitate the development of targeted, individualized approaches, thereby enhancing the effectiveness of strategic self-talk interventions for skill acquisition and performance enhancement.

In accordance with the two major types of self-talk cues, two broad clusters of mechanisms have been mostly forwarded within sport: attentional, focusing on the different domains and functions of attention (e.g., focused, selective, concentration), and motivational, focusing on cognitive, affective, and behavioral aspects of motivation (e.g., confidence, anxiety, effort) (Galanis & Hatzigeorgiadis, 2020). The attentional mechanisms have been identified as most pertinent for fine tasks and the early stages of learning. In recent years, a fair body of experimental studies has yielded consistent evidence for the effect of self-talk on attention. This support has been provided either directly, assessing attention as an outcome in non-sport computerized tasks (e.g., Gregersen et al., 2017), or indirectly, through studies exploring the effect of strategic self-talk in tasks with particular attentional demands (e.g., Galanis et al., 2026), implicitly attributing the effects to underlying changes in attentional functioning. Regarding the former, evidence indicates that strategic self-talk can assist in directing attention to appropriate stimuli (e.g., Gregersen et al., 2017), shifting attention across different attentional styles (e.g., Mallett & Hanrahan, 1997), and enhancing the appropriate focus of attention (Bell & Hardy, 2009). Regarding the latter, considerable attention has been devoted to the examination of the buffering effects of strategic self-talk on adverse conditions. This research has addressed a number of important attention-inhibiting factors, including ego depletion (Galanis et al., 2022), physical fatigue (Galanis et al., 2023), and external distractions (Galanis et al., 2018), and has supported that self-talk can help mitigate the detrimental effects of such factors. Summarizing the above, there is a reasonable body of evidence indicating that strategic self-talk exerts influence on attention through various functions that are contingent on the requirements of a given task and the nature of the task itself. Yet, to further advance this literature, the need to move beyond behavioral and self-report evidence has been identified (Ong, 2015). Towards this direction, adopting a more comprehensive psychophysiological approach to the study of self-talk mechanisms will help us understand what happens to the body that facilitates attention (Bellomo et al., 2020).

An important physiological indicator that has been receiving increased interest in recent years within the sport psychology literature is Heart Rate Variability (HRV) (Mosley & Laborde, 2022). The HRV metric reflects the dynamic influence of the vagus nerve on cardiac function (Laborde et al., 2017), providing insights into an individual’s physiological responses to physical and psychological stress (Stanfield, 2017). The vagus nerve has been identified as the primary nerve of the parasympathetic nervous system (Thayer et al., 2009), which is responsible for regulating several physiological processes, such as slowing down bodily functions and conserving energy. Accordingly, it has been linked with physiological changes, such as increased HRV, reduction in heart rate, and decreased myocardial contractility (Weissman & Mendes, 2021).

With regard to aiming sport tasks, higher parasympathetic activation has been linked to enhanced performance (Ortega & Wang, 2018). Psychological skills training, which has also been shown to facilitate learning and enhance performance in fine tasks (Hatzigeorgiadis et al., 2011; Rogers, 2006), provides a fertile ground to explore the impact of psychological techniques on the parasympathetic nervous system and its role in performance. In a recent study, Lee et al. (2023) investigated the impact of psychological skills training, encompassing techniques such as self-talk, imagery, relaxation, and goal-setting, on HRV. Their results provided indications that psychological skills training was linked to increases in parasympathetic activity, which was interpreted as an indicator of reduced arousal and anxiety. In a similar study, Galanis et al. (2025) examined the effects of strategic self-talk on HRV in a cohort of sport science students during an endurance cycling task. The findings revealed that during the final 2/5th of the 20-min task, a greater vagal suppression was observed for the control group, compared to the self-talk group. These differences in vagal modulations were attributed to the self-regulating effects of strategic self-talk, indicating a less effortful performance.

Integrating physiological indices with behavioral evidence in sport tasks would advance our understanding of the mechanisms through which self-talk facilitates learning and enhances performance (Dehais et al., 2020). Accordingly, the present study investigated the effects of a strategic self-talk intervention on skill acquisition in a fine golf-putting task requiring sustained attention, as well as its potential impact on autonomic nervous system activity, among novices. Golf is a closed and self-paced motor task, involving considerable cognitive processing; it requires focus of attention, eye-hand coordination, composure and self-control. Thus, it is an appropriate task for the investigation of the attentional self-talk mechanism, through behavioral and physiological assessment. Accordingly, it has which has been also used in previous research (Bell & Hardy, 2009; Bellomo et al., 2020). The primary goal, and the innovation of this study, was to explore changes in HRV as a function of the use of strategic self-talk in a fine task. Yet, for our results to hold value, the impact of the intervention on skill acquisition through task performance was also assessed. Therefore, we hypothesized that (a) performance of the two study groups would improve due to the learning effect introduced through the golf practice, but also that the performance improvements would be greater for the strategic self-talk group, and (b) the strategic self-talk group would display higher parasympathetic activation during the task.

## 2. Materials and Methods

### 2.1. Participants

Power analysis (G*Power 3.1.9.7, Düsseldorf, Germany) was conducted to estimate the number of participants required to achieve a minimum power of 0.80 at an alpha of 0.05, based on an estimated effect size of 0.48 (meta-analysis, Hatzigeorgiadis et al., 2011). The analysis indicated that 37 participants were required in total. To account for potential losses, 40 male sport science students were recruited. The mean age of the participants was 19.33 years (SD = 2.40). The participants in this study were required to be right-handed, as the golf club used was for right-handed people, and not to have any prior experience with golf putting. Participation was entirely voluntary, and participants were offered course credit in return for their participation.

### 2.2. Apparatus and Measures

#### 2.2.1. Performance

**Figure 1:**
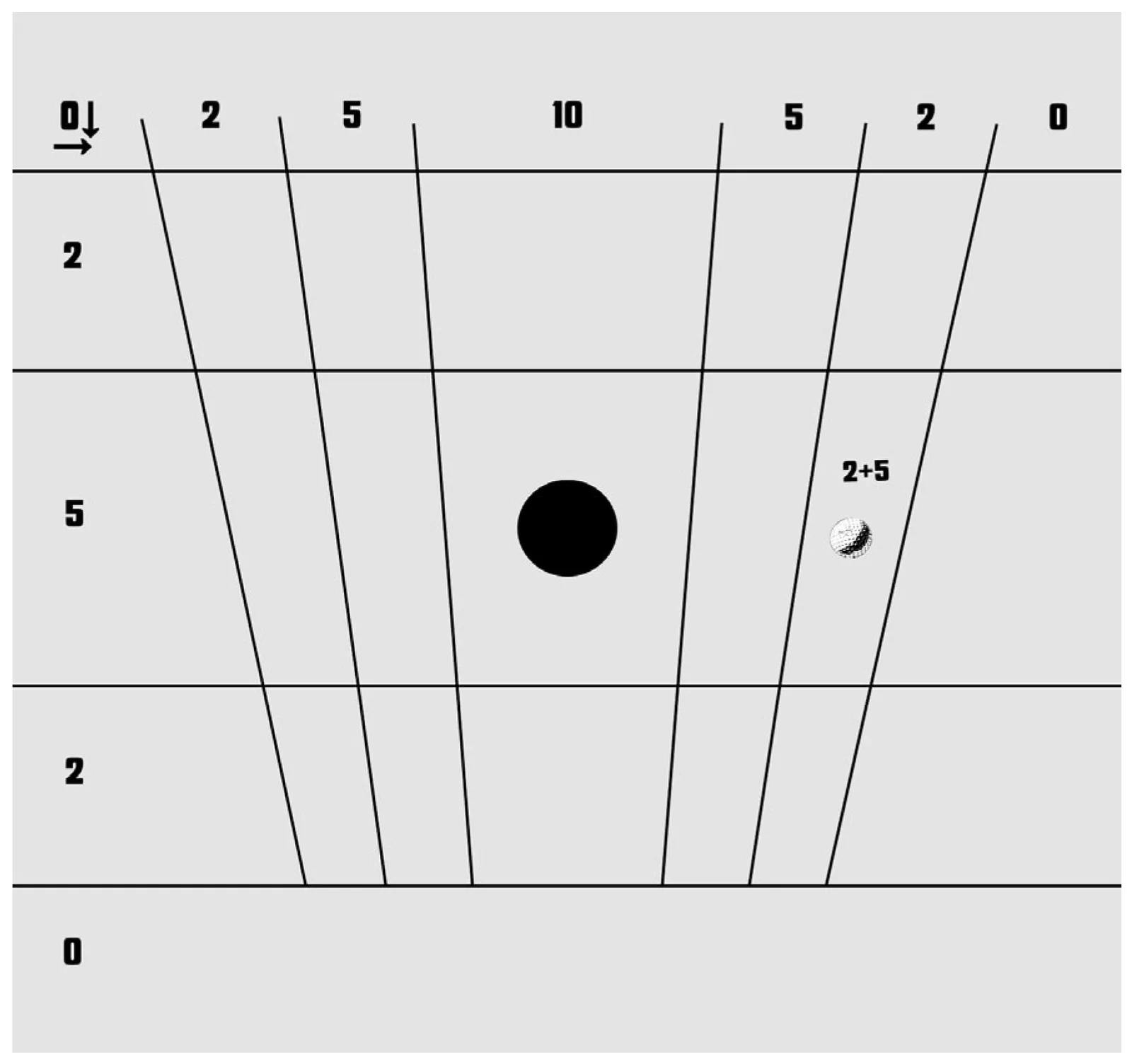
Scoring system used to measure putting performance. Note. Black circle: putting hole; white, shadowed circle and numbers: landed ball and respective score; arrows: pointing horizontal and vertical point system.

To evaluate putting performance, a custom-designed point system was devised (Figure 1) with the contribution of two golf experts. Considering that participants had no prior experience in golf, our aim was to provide a more nuanced and comprehensive assessment of performance, which would take into account not only the number of successful putts but also the trajectory and the distance for missed shots, and award points accordingly. The points awarded for each putt were the total of the vertical and horizontal scores as these appear in each distinct area in Figure 1. On-target balls were awarded 25 points. Performance was operationalized as the sum of the points achieved through 3 sets of 20 putting attempts. This performance approach aimed to provide a more accurate reflection of the participants’ learning improvement, with emphasis on accuracy and precision, while maintaining an ecological golf perspective. The putting task was conducted using an Amilla Mini-putter (for right-handed participants), on a specially designed grass strip that featured an uphill slope, from a distance of 2.5 m.

#### 2.2.2. Heart Rate Variability

To measure HRV, a Polar V800 connected to an H9 sensor on a chest strap was utilized. The vagus nerve has been identified as the primary nerve of the parasympathetic nervous system (Thayer et al., 2009), which is responsible for regulating several physiological processes, such as slowing down bodily functions and conserving energy. Two domains for assessing HRV are typically used: the time and the frequency domains, which are determined by the time intervals between each successive normal heartbeat. The time domain represents how a signal changes over time by measuring variance between individual heartbeats in milliseconds. The frequency domain represents how power is distributed throughout the time periods of a given rhythm by breaking them down into specific frequency bands. Accordingly, two vagally-mediated HRV indices associated with the central autonomic network, which is involved with executive functions (Thayer et al., 2009), thus particularly relevant for attentional processes, were employed in this study: the RMSSD and the HF (Mosley & Laborde, 2022). RMSSD is the principal time-domain metric for the assessment of vagal activity (Gullett et al., 2023), with higher values indicating greater parasympathetic activity. HF is the sole vagally-mediated among the frequency domain HRV indices, which records the amount of heartbeat variability within the 0.15–0.40 Hz frequency range, with higher values indicating greater parasympathetic activity.

### 2.3. Procedure

The study was designed and conducted in accordance with the ethical principles outlined by the Declaration of Helsinki, and with respect to the General Data Protection Commission. With regard to Transparency and Openness, the submitted article adheres to the Journal Article Reporting Standards (Appelbaum et al., 2018). We have reported how the sample size was determined, and all manipulations and measures in the study. The study was not registered; the data and the analyses are available upon request from the corresponding author. The study was approved by the institution’s ethics committee (ref: 2097).

A randomized, single-blinded, controlled trial was implemented. Participants were recruited through open calls and from the applicants; 40 were randomly selected. They were contacted and informed about the study procedures and were randomly assigned to control and experimental groups (1:1 ratio) using a simple randomization procedure (computer-generated random numbers). The experiment was completed in four sessions: baseline measurement, two training sessions, and final measurement. In an attempt to minimize the impact of external factors on the autonomic nervous system, the baseline and final measurements for each participant were conducted on the same day and hour, one week apart. The day before each measurement, participants were contacted and instructed to refrain from consuming alcohol, coffee, or energy drinks for three hours as well as avoiding large meals in the last three hours before the measurement. These requirements were self-reportedly confirmed by participants upon arrival for the assessment.

#### 2.3.1. Baseline Assessment

In the first session, participants received detailed information about the procedures and signed consent forms. They were then introduced to the golf putting technique, given instructions, and asked to perform 20 putts for familiarization, during which they received feedback and further instructions. Following the familiarization, the polar sensors were fitted, and participants began the baseline performance measurement, during which they received no feedback or instructions. Following each putt, a researcher situated close to the hole recorded the score and cleared the ball off the course, while a second researcher was placing a new ball on the 2.5 m mark. Three sets of 20 shots from 2.5 m were performed, with one-minute intervals between sets. The whole procedure lasted approximately 60 min, with each of the three performance sets lasting around 8 min.

#### 2.3.2. Intervention

The next two sessions involved practice. Both groups were asked to take 3 sets of 20 putts, from different distances (2 m, 2.5 m, and 3 m) in order to reduce the distance learning effect. A two-minute interval was allowed between the practice sets, during which participants of both groups were provided with technical feedback, according to a standard list of potential mistakes from the initial instructions. Participants of the experimental group received, in addition, a brief standard introduction to self-talk, and practiced with the use of different instructional self-talk cues based on the principles of the ST-IMPACT protocol for experimental research (Hatzigeorgiadis et al., 2014). In particular, following an explanation of what strategic self-talk is, they were introduced to the self-talk practice card to be used, which described the “what” (the actual cues), the “when” (the timing of the cue in relation to the execution), and the “why” (the message triggering the action) of the self-talk practice. On the first day of practice, they used a different instructional self-talk cue per set, which was assigned by the researcher, considering the major deficit based on the baseline assessment. On the second day of practice, they were asked to choose among a pool of suggested cues, including the ones practiced on the first day but also others of their liking, and ultimately to select the cue of preference for the final assessment. During the break between the sets, participants were also asked to reflect upon the used cues and their perceived fit for an effective implementation. The most commonly selected cues were ‘’Steady’’, ‘’In-line’’, and ‘’Smooth’’.

#### 2.3.3. Final Assessment

In the final measurement, the same procedures as the baseline were applied. Participants performed 20 warm-up shots, during which they received no feedback or instructions, and then proceeded to take 3 sets of 20 putts, with a one-minute break in between. Subsequently, a manipulation check was administered to the experimental group, asking participants to indicate on a scale from 1 (never) to 10 (all the putts) the degree to which they used their self-talk cue.

### 2.4. Data Analyses

HRV data were transmitted through the PolarFlow app 6.7.0 and subsequently analyzed using Kubios Standard version 3.5.0 (Biosignal Analysis and Medical Imaging Group, Department of Applied Physics, University of Eastern Finland, Kuopio, Finland). Artifact correction was conducted prior to the analysis, using the native beat correction algorithm with the threshold set to medium (0.3) (Laborde et al., 2017).

Performance and HRV data were screened for outliers; subsequently, the statistical assumptions for the planned analyses were tested. A *t*-test was conducted to examine potential differences in performance at baseline between the two groups. Accordingly, a multivariate analysis of variance was conducted to test for differences in HRV measures at baseline between the groups. Repeated measures ANOVA/MANOVA with one repeated factor (time) and one independent factor (group) were conducted to examine the effect of group and time on performance and HRV. Finally, a post-hoc additional analysis was conducted to further explore differences in HRV. This analysis involved a three-way repeated measures ANOVA with two repeated factors, time (pre, post) and putting set (1st, 2nd, 3rd), and one independent factor (group), which was followed up by two-way repeated measures ANOVAs with two repeated factors (time and set), for each group.

## 3. Results

### 3.1. Preliminary Analyses

Data inspection showed that there were no missing data. The screening of the data revealed outlying HRV values for one participant, due to bradycardia; the participant was therefore excluded from further analyses. The statistical assumptions for normality, homogeneity, and sphericity were met.

The independent samples *t*-test showed no baseline differences for performance between the two groups, t(37) = 0.602, *p* = 0.551. The one-way MANOVA revealed a non-significant multivariate effect, F(2, 36) = 0.35, *p* = 0.707, for the baseline HRV measures; accordingly, the univariate effects showed non-significant differences between the two groups: for RMSSD, F(2, 36) = 0.553, *p* = 0.462, and for HF, F(1, 37) = 0.610, *p* = 0.440. Inspection of the manipulation check questions showed that participants of the experimental group used the selected cues with great consistency (M = 9.67, SD = 0.66), thus reinforcing the efficiency of the strategic self-talk intervention.

### 3.2. Performance

**Figure 2:**
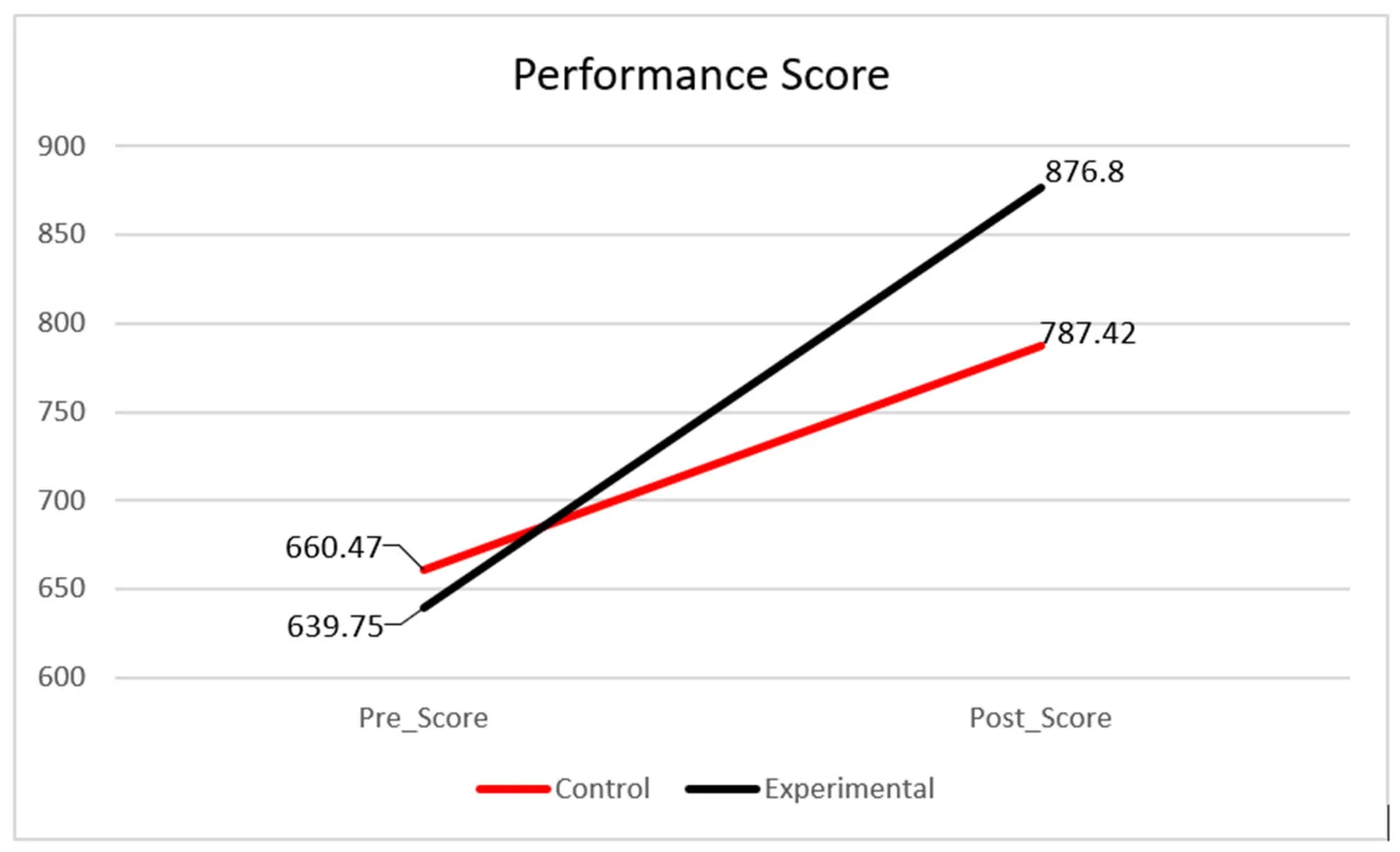
Changes in performance for the two groups from baseline to final assessment.

The repeated-measures ANOVA for performance showed a significant time by group interaction, F(1, 37) = 5.67, *p* = 0.022, partial η^2^ = 0.13. From the pairwise comparisons, it was revealed that both the control, F(1, 37) = 14.72, *p* < 0.001, and the experimental group, F(1, 37) = 54.01, *p* < 0.001, improved significantly from the baseline (M_ctr_ = 660.47, SD_ctr_ = 105.38, M_exp_ = 639.75, SD_exp_ = 109.18) to the final assessment (M_ctr_ = 787.42, SD_ctr_ = 122.72, M_exp_ = 876.80, SD_exp_ = 126.84). Nevertheless, it was also shown that (a) the effect for the experimental group (partial η^2^ = 0.59) was considerably higher than that of the control group (partial η^2^ = 0.29), and (b) in the final assessment, the experimental group had a significantly higher performance compared to the control group, F(1, 37) = 4.935, *p* = 0.033; yielding a large effect size, Hedge’s g = 1.05, SE = 0.34. The changes in performance are presented in Figure 2.

### 3.3. Heart Rate Variability

**Figure 3:**
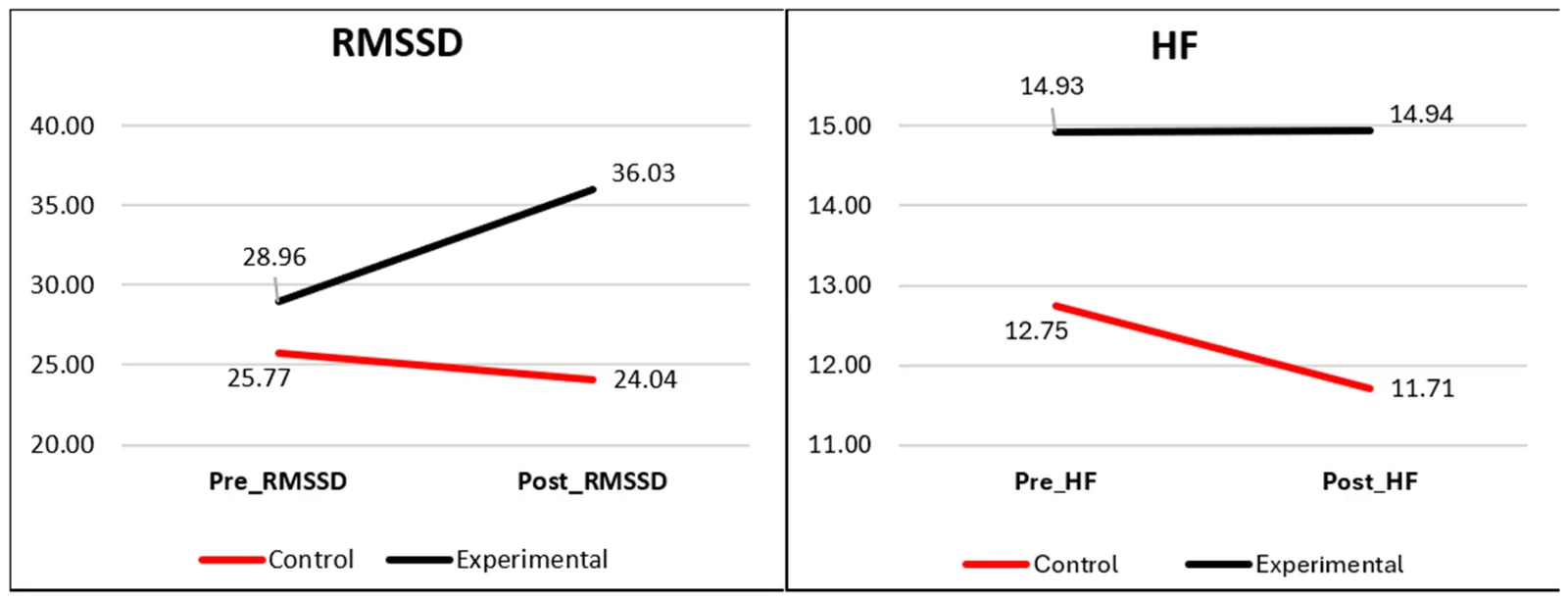
Changes in RMSSD and HF from baseline to final assessment for the two groups.

The repeated-measures ANOVA for HRV showed a non-significant multivariate effect, F(2, 36) = 1.98, *p* = 0.152; (partial η^2^ = 0.10); nevertheless, examination of the univariate effects provided some useful insights. In particular, for RMSSD the univariate analysis showed a marginally non-significant time by group interaction, F(1, 37) = 4.03, *p* = 0.052, partial η^2^ = 0.10; the examination of the pairwise comparisons showed that the experimental group showed increases in RMSSD (*p* = 0.027, partial η^2^ = 0.15), whereas no differences were recorded for the control group (*p* = 0.586). Non-significant effects were identified for HF, F(1, 37) = 0.334, *p* = 0.567, partial η^2^ = 0.001. The changes in the HRV indices are portrayed in Figure 3.

**Figure 4:**
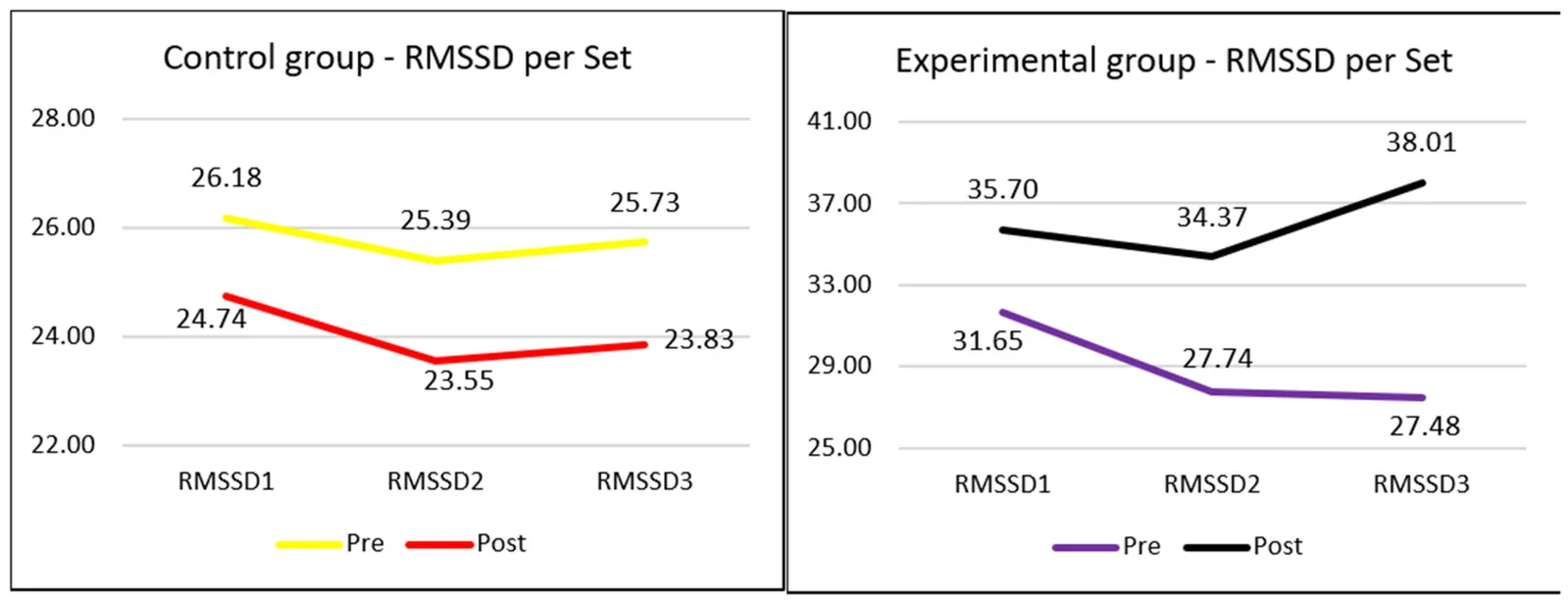
Changes in RMSSD per time per set for the control and experimental group.

To further explore the effect identified for RMSSD in relation to the length of the experimental task that included three performance sets, a three-way repeated measures ANOVA with two repeated factors, time (pre, post) and putting set (1st, 2nd, 3rd), and one independent factor (group) was conducted. The analysis yielded a marginally non-significant multivariate three-way interaction, F(2, 36) = 3.021, *p* = 0.061, partial η^2^ = 0.14. To further examine this three-way interaction, two-way ANOVAs with two repeated factors, time (pre, post) and putting set (1st, 2nd, 3rd), were conducted separately for each group. The analysis for the control group did not exhibit a significant multivariate interaction effect, F(2, 17) = 0.076, *p* = 0.927, partial η^2^ = 0.01, whereas a marginal interaction was identified for the experimental group, F(2, 18) = 3.476, *p* = 0.053, partial η^2^ = 0.28. Examination of the pairwise comparisons showed that while for the control group no differences in RMSSD were recorded between the three sets across baseline and final assessment, for the experimental group, RMSSD increased from set 2 to set 3 in the final assessment, (*p* = 0.019, partial η^2^ = 0.26), resulting also in a significant difference for set 3 between baseline and final assessment with a large effect (Hedge’s g = 0.949, SE = 0.11). Descriptive statistics for RMSSD across time and set for the two groups are presented in Table 1; the visual patterns are portrayed in Figure 4.

## 4. Discussion

The pivotal purpose of this study was to explore the potential impact of strategic self-talk on the functioning of the autonomic nervous system, attempting to provide physiological evidence for the attentional interpretation of the strategic self-talk effectiveness for skill acquisition. A randomized controlled trial was implemented, where performance and HRV were measured before and following a strategic self-talk intervention. Through the self-talk practice, participants were gradually given input on the use of the self-talk cues with the intention to maximize the effectiveness of the training. Consistent with the literature, the self-talk intervention had a considerable effect on skill acquisition among novices. Furthermore, the results from the HRV analyses, despite the lack of multivariate significance, yielded interesting patterns of change for RMSSD, which provided indications for a less effortful physiological response from the experimental group during task execution, and in particular in the latest stages of the task.

As anticipated, both the control and experimental groups displayed improved performance, due to the learning effect induced by the two practice sessions on a task that was initially unfamiliar to the participants. Nonetheless, the experimental group showed a more substantial improvement and a significantly better performance in the final assessment compared to the control group. This adds further evidence for the effectiveness of self-talk interventions in facilitating learning and enhancing sports task performance, in particular for fine tasks among novices.

The analysis of the HRV variables, measured as an average for the duration of the experiment, revealed a non-significant multivariate effect. However, inspection of the univariate tests revealed a marginally non-significant difference with a large effect in RMSSD values, with the pairwise comparisons showing a significant increase from baseline to final assessment for the experimental group. These results provided limited but intriguing indications that the self-talk group exhibited an increase in the activation of the parasympathetic system. Cognitive, affective, and physiological regulation have been associated with vagally-mediated cardiac function (Thayer & Brosschot, 2005). Accordingly, it can be postulated that in our final assessment, the rising trends in RMSSD for the instructional self-talk group may reflect better autonomic resource allocation and regulation, which produced better task performance. These findings appear consistent with existing physiological research on aiming tasks, which have found that higher performance is associated with higher RMSSD values (Ortega & Wang, 2018).

Follow-up analysis yielded further interesting evidence, as it was identified that for the strategic self-talk group, RMSSD values significantly increased and differed from those of the control group during the last golf-putting set. Comparable effects have been noticed by Galanis et al. (2025) in a strategic self-talk intervention in a cycling task. In that study, it was reported that participants of the strategic self-talk group showed a smaller decline of parasympathetic activation compared to the control group during the last 8 min of the 20 min trial. Studies in the self-talk literature have supported that strategic self-talk can mitigate the adverse effects evoked by attention-challenging conditions, such as distraction (Galanis et al., 2018), physical exertion (Galanis et al., 2023), and, mostly relevant to this study, ego-depletion (Galanis et al., 2022). Regarding the latter, the study of Galanis et al. (2022) provided evidence that strategic self-talk assisted performance in two experiments involving golf-putting following ego-depletion manipulations. Considering the buffering effect of strategic self-talk, the authors forwarded an attentional interpretation, suggesting that when depleted, self-talk can help individuals maintain attention processing efficiency (Bortoli et al., 2012). Bearing in mind the duration of the task (approximately 60 min) and the mental load that was required to maintain attention and composure for the golf putting task in the present experiment, it is reasonable to assume that the novice participants experienced some levels of depletion. The identified self-talk effect for RMSSD in the later stages of the assessment seems to coincide with the above findings from Galanis et al. (2025). While RMSSD and HF are highly correlated (Kleiger et al., 2005), in our experiment HF didn’t follow the same pattern. This could be due to the influence respiration has on the HF index (Eckberg & Eckberg, 1982). In the experiment of Galanis et al. (2025) where an endurance task was used, RMSSD and HF showed more compatible trends. This could possibly be attributed to the more efficient regulation of respiration rhythm, which was not challenged in our golf-putting task.

Notwithstanding the marginally non-significant multivariate effect, considering the univariate effects and in particular the considerable effect size that emerged, the findings provide valuable exploratory trends for the attentional self-talk mechanism, postulating that strategic self-talk can mitigate the impact of ego-depletion through the facilitation of attentional processing. The self-talk literature has provided considerable evidence for the attentional effects of self-talk as a mechanism explaining its effectiveness for task performance. Attention-inhibiting factors, such as ego depletion and physical exhaustion, reduce available attentional resources and constrain the processing of relevant cues (Hagger et al., 2010; Janelle et al., 1999). Attempting to explain the facilitating effects of self-talk on attention-challenging tasks, Galanis and Hatzigeorgiadis (2020) argued that self-talk can help counter such detrimental effects by preserving or renewing attentional resources. Such attention effects can reduce the impact of adverse conditions, such as physical exertion and ego depletion, benefit attentional processes, and, subsequently, performance.

From a psychophysiological perspective, attentional regulation has been linked with working memory capacity; when learning a new task, the bigger the load of information that needs to be stored and processed, the greater the mental effort that is required to perform the task (Baddeley & Hitch, 1974). On the one hand, self-talk as a cognitive strategy has been shown to direct attention to task-relevant cues and decrease task-irrelevant thoughts, thus reducing the load of information to be processed by the working memory (Hatzigeorgiadis & Galanis, 2017). On the other hand, working memory and HRV share a common neural basis through the central autonomic network (Thayer et al., 2009). In a study investigating the links between HRV and cognitive workload, participants with higher HRV displayed a more solid and stable performance in a working memory task, compared to participants with lower HRV (Hansen et al., 2008). The authors postulated that higher HRV was associated with better self-regulation and adaptability. Thus, it can be speculated that by reducing cognitive load and saving cognitive resources, self-talk can bring increases in parasympathetic activity. Such increases in the vagal tone can subsequently facilitate the progress of skill acquisition, in particular in a task like golf; a motor task, characterized by sustained focus of attention, eye-hand coordination, composure and self-control, thus requiring considerable cognitive processing at the skill acquisition stage.

### 4.1. Considerations and Future Research Directions

Two important considerations should be taken into account when interpreting the present findings. First, the lack of multivariate significance in the original HRV analysis. Even though the performance effect was considerable, and the analysis was well-powered, the HRV changes were only significant at the univariate level. Having acknowledged that, the present results provide new evidence for subsequent power analyses involving the effects of strategic self-talk on HRV. Evidently, the interpretation of these findings should be cautious; yet the results from the follow-up analysis enhance our confidence in this cautious interpretation. Second, the interpretation from the follow-up, set-by-set, analysis regarding the ego-depletion effect should also be cautious, as ego-depletion was not assessed but rather assumed due to the length of the experimental procedure.

In addition, the sample boundaries of the study should be acknowledged. Participants were males with no prior experience in golf. The gender choice was made to enhance the homogeneity of the sample but also to avoid inconsistent HRV data due to the potential impact of the menstrual cycle on the autonomic nervous system (Brar, 2015). For scientific and ethical reasons, female students were invited to a subsequent experiment. Furthermore, the selection of participants with no prior experience in golf in our investigation limits the external validity of the study and restricts any implication to novices or individuals at the early stages of skill acquisition. Finally, although the exclusion of left-handed participants does represent a major source of bias, it should be acknowledged as a boundary of our study.

With regard to the HRV assessments, even though the baseline and final assessment for each participant took place on the same day and time, one week apart, participation times across individuals varied; this may have influenced HRV recordings via diurnal variation. Subsequently, future studies should consider standardizing participation times. Moreover, our research protocol included the assessment of tonic HRV only during task execution, as self-talk takes place in a short and particular timeframe, before the execution. Subsequent studies recording HRV also before (resting) and following the actual task execution would allow us to assess phasic HRV, i.e., reactivity and recovery HRV, which would provide a more comprehensive approach to the self-regulatory mechanisms of the HRV.

Although HRV is a reliable indicator of psychophysiological states, additional physiological measures should be incorporated to deepen our understanding of self-talk mechanisms. In particular, evaluating brain activity concurrently with HRV would yield more informative data regarding psychophysiological states involving attention. Moreover, the utilization of eye-tracking technology may also prove advantageous when measuring psychological influences on aiming tasks, as eye gaze, quiet eye phenomenon, and pupil dilation have all been linked to attention and performance.

### 4.2. Conclusions

The present study explored the impact of strategic self-talk on the skill acquisition of a fine task, and in parallel, changes in heart rate modulations. The findings confirmed the facilitating effects of strategic self-talk on skill acquisition. In addition, the results yielded interesting patterns about the potential impact of strategic self-talk on HRV modulations, providing indications for an attentional interpretation of the self-talk effects indicating a less effortful functioning, in particular towards the latest stages of the task when participants were likely depleted. The study adds to the limited existing literature examining self-talk mechanisms through a psychophysiological perspective, provides valuable evidence and relevant directions to be explored, and encourages further multidisciplinary research for the understanding of the self-talk phenomenon.

## Figures and Tables

**Table 1:** Pairwise comparisons between and across sets for baseline and final assessment for RMSSD values.

		Control			*p*		Experimental			*p*	
		Set 1	Set 2	Set 3	1vs2	2vs3	Set 1	Set 2	Set 3	1vs2	2vs3
Baseline	Mean SD	26.18 9.61	25.39 8.87	25.73 9.14	0.35	0.65	31.65 18.70	27.74 15.68	27.48 15.68	0.001	0.76
Final	Mean SD	24.74 10.48	23.55 9.16	23.83 8.08	0.17	0.59	35.70 32.02	34.37 27.21	38.01 29.29	0.62	0.05
*p*		0.51	0.34	0.33			0.18	0.10	0.02		

## Data Availability

The data presented in this study are available on request from the corresponding author due to privacy reasons.

## Abbreviations

The following abbreviations are used in this manuscript:

HRV	Heart-Rate Variability
RMSSD	Root Mean Square of Successive Differences
HF	High-Frequency
